# A tale of 2 gasses, 1 regulator, and cholesterol homeostasis

**DOI:** 10.1371/journal.pbio.3002401

**Published:** 2023-11-22

**Authors:** Nicole M. Fenton, Andrew J. Brown

**Affiliations:** School of Biotechnology and Biomolecular Sciences, UNSW Sydney, Sydney, Australia

## Abstract

There is a growing appreciation of the wide-ranging effects of carbon dioxide on transcriptional regulation and metabolism. This Primer explores a study that provides the first link between carbon dioxide and the master transcriptional regulator of cholesterol homeostasis.

## Teaser

There is a burgeoning appreciation for the wide-ranging effects of carbon dioxide on transcriptional regulation and metabolism. Here, Bolshette and colleagues provide the first link between carbon dioxide and the master transcriptional regulator of cholesterol homeostasis.

Two gaseous waste products perform what must be the greatest atomic shuffle in life. Oxygen exhaled by plants and other photosynthetic lifeforms power aerobic metabolism in animals like us. With each breath, a little carbon escapes [1], lifted on wings of oxygen. This invisible gas was first dubbed “fixed air,” but we know it better as carbon dioxide (CO_2_). Given the centrality of CO_2_ to our metabolism, perhaps it is not surprising roles are emerging for this previously dismissed waste product in key metabolic pathways, including those involving cholesterol. In this issue of *PLOS Biology* [2], Bolshette and colleagues link CO_2_ levels to the master transcriptional regulator of cholesterol homeostasis, Sterol Regulatory Element Binding Protein-2 (SREBP2).

**Fig 1:**
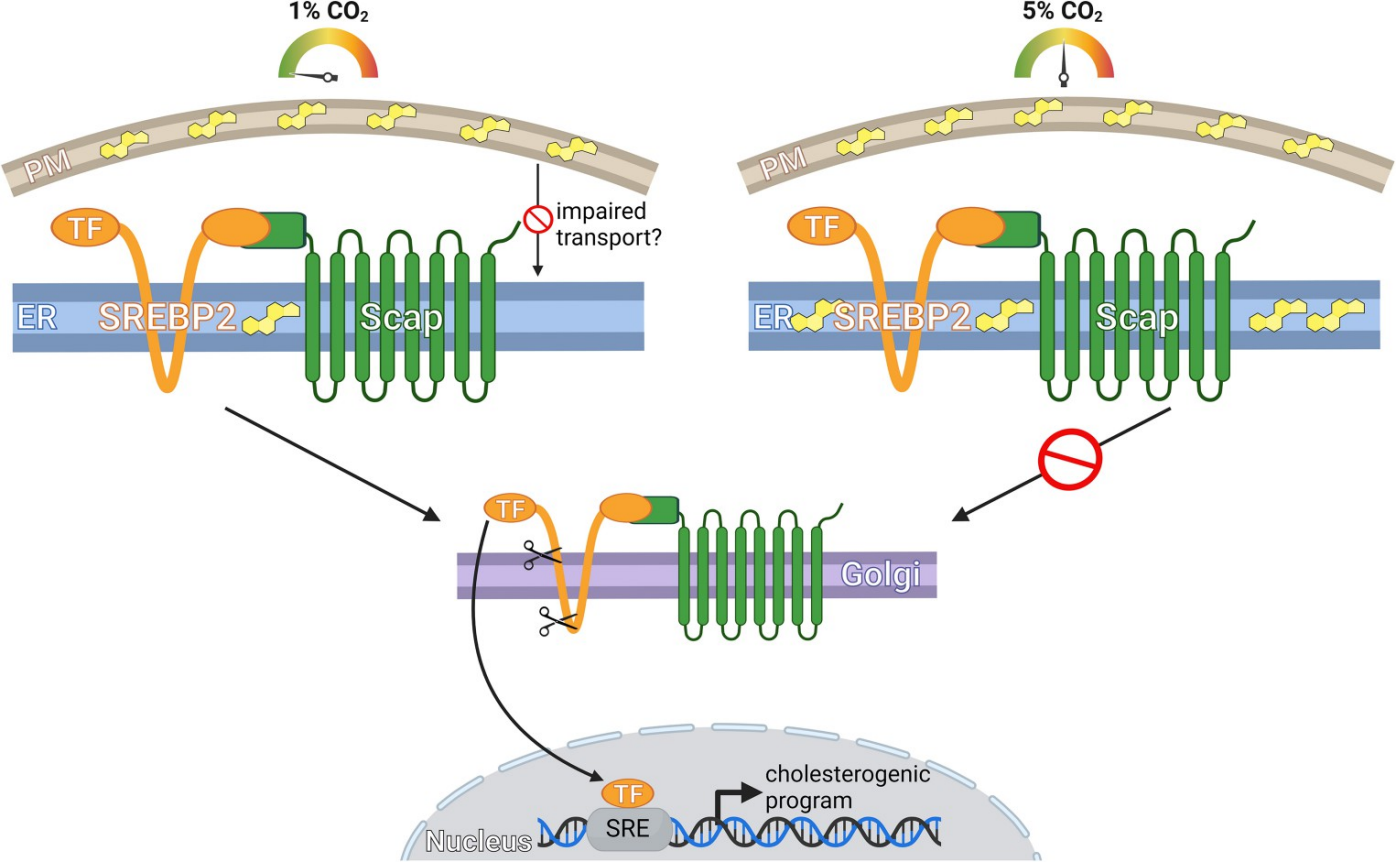
A simplified model of how varying CO_2_ levels affect SREBP2 activation. Low CO_2_ (1%) results in less cholesterol (yellow structure) in the ER allowing SREBP2 to be transported to the Golgi for proteolytic cleavage to release the active TF. However, at physiological CO_2_ levels (5%), transport from the ER to the Golgi is inhibited. One possibility is that low CO_2_ disrupts cholesterol trafficking between the ER and PM. Created with BioRender.com.

Our cholesterol concentrations are exquisitely regulated to ensure levels are sufficient yet not toxic. Multiple layers of regulation occur to achieve cholesterol homeostasis, including coordination of a transcriptional program through the elegant SREBP2/Scap pathway [3]. SREBP2 begins life in the membranes of the endoplasmic reticulum (ER). Insufficient cholesterol is sensed by Scap within the ER membranes to allow transport of SREBP2 to the Golgi for proteolytic activation and subsequent induction of genes involved in cholesterol uptake and biosynthesis (**Fig 1**). When cholesterol levels are sufficient, SREBP2 remains within the ER, switching off the cholesterogenic transcriptional program.

Bolshette and colleagues [2] discovered a reduction in CO_2_ results in the activation of SREBP2 through decreased levels of cholesterol in the ER via an unknown mechanism. Firstly, using cultured murine fibroblasts as their main cell model, they identified genes with altered transcription in response to changing CO_2_ levels. Notably, genes involved in cholesterol biosynthesis and related processes were largely up-regulated or suppressed in response to low (1%) versus normal (5%) or high (10%) CO_2_ exposure, respectively. This effect was largely independent of pH changes known to occur with varying CO_2_ concentrations. The up-regulation of the cholesterogenic program by low CO_2_, the focus of this study, was generalisable to other cell types (notably liver and muscle), although not to primary adipocytes.

SREBP2 was identified as one of the top transcriptional regulators in response to low CO_2_. Indeed, immunoblot analysis showed the cleaved, nuclear form of SREBP2 accumulated within only 2 h of low CO_2_ exposure. SREBP2 activates transcription of target genes by binding to sterol regulatory elements (SREs) within promoters of genes. Employing an SRE luciferase reporter assay, Bolshette and colleagues [2] observed increased luminescence over time under low CO_2_ conditions in cells expressing a wild-type (but not mutated) SRE reporter. This effect was reversible, luminescence fading away when CO_2_ levels returned to 5% from 1%.

The translocation of SREBP2 from the ER to the Golgi prior to entering the nucleus is an essential step in SREBP2 activation. Both knockdown of *Scap* by siRNA and pharmacological inhibition of ER to Golgi trafficking of SREBP2/Scap (using fatostatin) prevented the SREBP2-mediated responses under low CO_2_. SREBP2 activation is largely driven by changes in ER cholesterol levels. Low CO_2_ did not change total cell cholesterol levels. However, ER levels were depleted, determined after a rigorous cell fractionation protocol [4]. Consistent with this finding, addition of sterols (cholesterol or an oxysterol) inhibited activation of SREBP2 under low CO_2_ conditions.

Another recent study [5] reported low O_2_ (hypoxia) shuts down the SREBP2 pathway by promoting the ubiquitination and degradation of this transcription factor (TF). However, here the mechanism is very different since Bolshette and colleagues [2] found low CO_2_ did not affect SREBP2 stability, but rather, low CO_2_ activates SREBP2 target genes by reducing cholesterol in the ER. Accordingly, Bolshette and colleagues [2] found that further depleting cell cholesterol by using cyclodextrin blunted the effect of low CO_2_.

But precisely how CO_2_ levels influence ER cholesterol levels remains the big question. The fact total cell cholesterol levels remained unchanged after acutely dropping CO_2_ levels, but ER cholesterol levels are decreased, suggests the residual cholesterol is trapped elsewhere, perhaps the plasma membrane (PM) where most cell cholesterol resides [4]. Certainly, the phenotype of reduced ER but unchanged total cell cholesterol is reminiscent of a block in PM to ER cholesterol transport observed by others [4]. One scenario is low CO_2_ levels destabilize a transporter [6] shuttling cholesterol between the PM and the ER. Increased CO_2_ may lead to a posttranslational modification (carbamylation) of lysine residues [7], with the potential to block ubiquitination sites targeting the transporter for degradation. But of course, there are a myriad of other possibilities. Determining if cholesterol derived from lipoproteins also blocks the effect of low CO_2_ would help better define the transport defect, considering lipoprotein-derived cholesterol meanders through the endo-lysosomal pathway.

It is unclear whether coupling of CO_2_ to cholesterol synthesis may be an adaptive or maladaptive response. Considering cholesterol impedes CO_2_ transport across membranes, perhaps an increase in cholesterol synthesis helps maintain intracellular CO_2_ levels [8].

What links CO_2_ and cholesterol homeostasis in (patho)physiology? Indeed, it is unclear if CO_2_ itself or bicarbonate is the active agent, considering the 2 are in rapid equilibrium [9]. Moreover, the physiological relevance of 1% CO_2_ is questionable, considering CO_2_ levels in blood normally range from 4% to 6% (estimated from 35 to 45 mmHg [10]). However, the general finding is likely still applicable considering Bolshette and colleagues [2] found the effect of CO_2_ was graduated from 1% to 10% CO_2_. The idea that the response is tuneable across the physiological and pathophysiological range has implications for diseases associated with low (e.g., early asthma) [9] and high CO_2_ (e.g., chronic obstructive pulmonary disease) [11].

To conclude, 2 recent studies have implicated each of the key respiratory gasses in regulating cholesterol homeostasis via SREBP2. Analogous to the tussle between O_2_ and CO_2_ for hemoglobin [7], these 2 gasses have opposing effects on SREBP2 activation and hence regulation of cholesterol biosynthesis genes. Low O_2_ prevents SREBP2 activation [5], whereas low CO_2_ increases activation [2]. While the O_2_- and CO_2_-dependent responses and mechanisms appear completely distinct, the signals may cross talk or compete physiologically. Of note, cholesterol biosynthesis is highly O_2_-intensive [12] and one of the few biosynthetic processes that also generates CO_2_. So, involvement of these 2 gasses in cholesterol homeostasis is perhaps unsurprising. But understanding how CO_2_—a carbon atom lifted on wings of oxygen—works its “magic” to help control cholesterol should inspire the field with a breath of fresh air.
